# New Appliances & Things Medical

**Published:** 1911-04-29

**Authors:** 


### New Appliances & Things Medical.

### LYMPHOID COMPOUND.

(The British Organotherapy Co., Ltd., 43 Carlton House, Lower Regent Street, S.W.)

The formula of this preparation, given on every box, shows that it consists of lymphatic-gland extract, glycerophosphates of iron, sodium, and calcium, and strychnine. It is therefore a strong tonic, and it has been very highly recommended by those who have used it in cases of nervous debility following acute diseases, in neurasthenia, and in most organic and functional affections of the nervous system. The preparation is put up in neat gelatine capsules, and the dose is one capsule before meals. Full literature and samples are supplied on application to the Company at the above address.

